# Differences in the Levels of the Selected Phytoestrogens and Stable Isotopes in Organic vs. Conventional Hops and Beer

**DOI:** 10.3390/foods10081839

**Published:** 2021-08-09

**Authors:** Jelena B. Golubović, Ester Heath, Iztok Jože Košir, Nives Ogrinc, Doris Potočnik, Lidija Strojnik, David Heath

**Affiliations:** 1 Jožef Stefan Institute, 1000 Ljubljana, Slovenia; ester.heath@ijs.si (E.H.); nives.ogrinc@ijs.si (N.O.) doris.potocnik@ijs.si (D.P.); lidija.strojnik@ijs.si (L.S.); david.heath@ijs.si (D.H.); 2 Jožef Stefan International Postgraduate School, 1000 Ljubljana, Slovenia; 3 Institute for Hop Research and Brewing, 3310 Žalec, Slovenia; iztok.kosir@ihps.si

**Keywords:** beer, hops, organic, liquid chromatography coupled to tandem mass spectrometry (LC-MS/MS), phytoestrogen, stable isotopes, isotope ratio mass spectrometry (IRMS)

## Abstract

Xanthohumol (XN), isoxanthohumol (IX) and 8-prenylnaringenin (8-PN) are important prenylflavonoids present in hops with potential beneficial properties. In this study, we examined differences in the content of XN, IX and 8-PN in hops and beer produced under organic and conventional production regimes. A An ultra-high performance liquid chromatography coupled to tandem mass spectrometry (UHPLC-MS/MS) method for analysing XN, IX and 8-PN in hops and beer was developed and validated, with LOQ ranging from 0.5 to 10 ng/mL. Finally, we examined ^15^N/^14^N and ^12^C/^13^C isotope ratios in the hops and beer using isotope ratio mass spectrometry (IRMS). The results show no statistically significant difference in the content of the selected prenylflavonoids between organic and conventionally produced hops and beer—in the whole sample group, as well as between the matched pairs. Stable isotope analysis indicated that only *δ*^15^N values are statistically higher in organically produced hops and beer. However, the differentiation according to the type of production could not be made solely based on the *δ*^15^N signature, but it could be used to provide supporting evidence.

## 1. Introduction

Beer production typically includes only four ingredients: water, cereal grain (e.g., barley, wheat or corn), yeast and hops, and it is the female inflorescences (cones) of the hop plant (*Humulus lupulus* L.) that are used to give beer its characteristic bitterness and floral, fruity or citrus flavours and aroma. Hop consists of many different organic compounds, which can be classified as primary and secondary metabolites based on their role in the plant’s life [1]. While primary metabolites have roles associated with nutrition, growth, development and reproduction, secondary metabolites are mainly involved in ecological interactions, including defence and competition [1].

**Figure 1:**
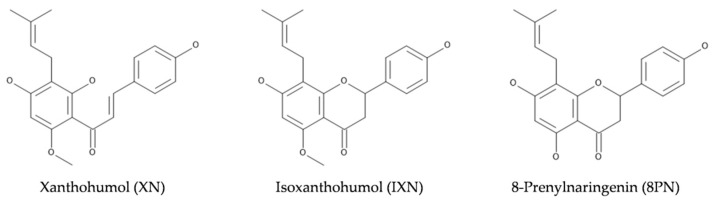
Chemical structures of the selected prenylflavonoids.

Among the secondary metabolites are polyphenolic compounds such as chalcones and flavanones. Examples include xanthohumol (XN), which is unique to hops, isoxanthohumol (IXN) and 8-prenylnaringenin (8PN), and are of particular interest to the scientific community because of their bioactivity [2,3]. They are members of the prenylflavonoid family of compounds, and contain the common prenyl (3-methyl-1-buten-3-yl) moiety (Figure 1). According to in vitro and in vivo studies, 8PN is a potent phytoestrogen with a strong binding affinity for estrogen receptors, particularly ERa, with an EC50 equal to 1.5 μg/L or 4.4 nM [3]. Isoxanthohumol, the predominant prenylated flavonoid in beer, is also considered pro-estrogenic since it can be O-demethylated to 8-prenylnaringenin by the action of cytochrome P450 or by intestinal microflora [3].

The estrogenic activity of hops was initially discovered due to female hop-pickers frequently reporting menstrual disturbances, and since then, the question about the estrogenic effect of beer has been asked many times. For example, Sauerwein and Meyer [4] concluded that beer’s estrogenic activity is equivalent to only a few µg estradiol/L, and an adult would need to drink more than a thousand litres of beer a day to experience adverse feminisation [4]. However, the authors conducted only in vitro studies and did not consider the effects of metabolism, such as the intestinal transformation of IXN to 8PN or how other ingredients affect the metabolism of phytoestrogens [3].

Preclinical animal studies and clinical trials suggest that phytoestrogens may be beneficial in treating climacteric symptoms in perimenopausal women or prevent and treat osteoporosis in postmenopausal women, but more extensive clinical trials are necessary to confirm their benefits [5]. Xanthohumol, the predominant prenylflavonoid in the hop, also shows high antioxidant activity, thereby having a potentially beneficial role in preventing and treating cancer, liver injury, neurodegenerative processes and skin inflammation. However, XN is only a minor component in beer due to its thermal isomerisation during the brewing process [5,6,7].

### 1.1. Organic Production

Organic production has many advantages, especially within the ecological and health domain, such as sustainable agriculture, by improving soil quality and fertility, reducing pollution and encouraging agricultural waste recycling. Even though the absence of pesticides and other chemicals contributes to better human health, the question of whether organic crops are more abundant in nutrients remains. Proteomic studies have shown that different proteomes of organically produced crops due to organic farming systems can affect mineral uptake (nutrient utilisation) and metabolic processes in crop plants compared to conventionally produced crops [8].

The assumption that there is a higher content of specific secondary metabolites in organically produced crops than in conventionally produced crops is supported by botanical theory, as De Keukeleire et al., writes “organic plants need to have a much more intricate arsenal of compounds for defence against pests than plants that are protected by a variety of fertilisers and spraying agents” [9]. Studies comparing secondary metabolites in plants grown under different production regimes, for example, using systemic meta-analyses accept [8], reject [10] or partially confirm this hypothesis, i.e., for the defence-related secondary metabolites [11]. Many factors can explain these differences, such as the diversity of compounds and crops, differences in how the results are interpreted, the inclusion of studies published before the EU organic farming regulation was introduced, and the different regulations between Europe and America [12]. Finally, a strong publication bias must not be omitted, i.e., the publication of only affirmative results [13,14].

So far, only one comparative study looks at the content of secondary metabolites, including prenylflavonoids in hops produced organically and conventionally [9]. The authors investigated the synthesis of secondary metabolites (XN among them) in three organic and conventionally produced hop varieties: First Gold, Admiral (A) and Wye Challenger. The authors also took into account climate and year of production. Interestingly, First Gold showed enhanced secondary metabolite production when grown organically, a finding that was consistent throughout the three harvest seasons. The two other hop varieties, Admiral (A) and Wye Challenger, did not show any uniform pattern and, the results are inconsistent [9]. At present, we are aware of no published study that has looked at the difference in prenylflavonoids between organically and conventionally produced beers. In addition, the ability to distinguish between organic (where at least 95% of the ingredients must be organically produced) from conventionally produced beer remains a significant challenge. In this regard, prenylflavonoids may act as biological markers of the production regime of hop and beers, especially if combined with the use of stable isotopes.

### 1.2. Stable Isotopes

Among the lighter elements having two or more stable isotopes (H, C, N, O, S), the stable isotopic composition of nitrogen (*δ*^15^N values) has been used to differentiate between conventional and organically produced crops [15,16,17,18].

In this context, the use of isotopes is based on the fact that synthetic fertilisers, allowed in conventional and integrated agriculture practice, have *δ*^15^N values around 0‰ [19], but the values of organic manures lie between +2 to +10‰ [20,21]. Thus, organic plant products vary within a range of *δ*^15^N values of +0.3 to +14.6‰, while conventional plant products range from negative to positive values, i.e., −4.0 to +8.7‰ [22]. Literature data presented by Mantha et al. [15] indicate that the mean *δ*^15^N value of organically grown plants of 7.7 ± 4.4‰ (median = 7.2‰), while it was 2.8 ± 2.3‰ (median = 3.0‰) for plants fertilised inorganically, resulting in a *δ*^15^N_org-conv_ of 4.2‰. The stable isotopes of carbon (*δ*^13^C values) can also act as an indicator of crop management practice. During photosynthesis, fertilisation performed in conventional systems involves a higher N supply to the plant than in organic fertilisation and may modify the C isotope signal due to its effects on stomatal conductance [16]. However, most literature studies are concerned with source identification, and it appears that there are no published studies on the type of production regime, i.e., conventional versus organic beer. For example, *δ*^13^C values were applied in studies to identify the geographical and botanical source of plants used for beer production [17,18,23,24], rather than management practice discrimination.

In this study, the contents of XN, IXN and 8PN in beer brewed in-house from organic, and conventionally grown hops samples were determined to obtain an insight into the transformation of these prenylflavonoids during the brewing process, i.e., whether the content of the selected prenylflavonoids in hops is reflected in the content of the beer. Given the selected activity of prenylflavonoids, the results could prove beneficial for the botanical dietary supplement industry or beer industry in changing beer consumption habits, i.e., increasing organic beer production and consumption using a higher number of varieties to obtain statistically significant results. Moreover, because of the lack of data regarding the differences between organic and conventional beers, this study set out to use a stable isotope approach (*δ*^15^N and *δ*^13^C) together with the XN, IXN and 8PN content to distinguish between conventionally and organically produced hops and conventionally brewed beers and beers labelled as organic on the market, i.e., to act as markers to detect organic production frauds.

## 2. Materials and Methods

### 2.1. Chemicals, Solvents and Materials

Isoxanthohumol (≥99%) was purchased from Sigma Aldrich GmbH, while xanthohumol (≥98%) and 8-prenylnaringenin (≥ 98%) were purchased from Enzo Life Sciences, Farmingdale, New York. Eriodyctiol (≥ 99%) and 3,4,2’,4’,6’-pentahydroxychalcone(≥99%) were purchased from Extrasynthese Lyon, France. Stock solutions of all reference standards were prepared in HPLC grade methanol (J. T. Baker, Deventer, The Netherlands). The same solvent was used for the extraction. Acetonitrile (ACN) was used as the organic part of the mobile phase (LC-MS grade, J. T. Baker, Deventer, Netherlands). Both formic acid (FA) used for SPE (>98% purity) and FA (LC-MS grade) used as a mobile phase modifier were purchased from Sigma Aldrich (Darmstadt, Germany).MilliQ (18.2 MΩ·cm) water was obtained using a Direct-Q^®^Water Purification System (Merck, Darmstadt, Germany). Strata-X 33 µm polymeric reversed phase cartridges (60 mg/3 mL) were purchased from Phenomenex (Torrance, CA, USA). Before injection into the LC-MS system, the samples were filtered through 0.2 µm regenerated cellulose (RC) membrane syringe filters (Phenomenex, Torrance, CA, USA). Hops and beer samples were freeze-dried in a GAMMA 1-16 LSCPlus freeze dryer (Martin Christ Gefrierungstrochnungsanlagen GmbH, Osterode am Harz, Germany).

### 2.2. Sampling

Samples of hop pellets were purchased from Hopfen und mehr, Neukirch, Germany; Hopfen der Welt, Ellingen, Germany; donated by Dr Biendl or collected directly from Slovenian hop growers by the *Slovenian Institute of Hop Research and Brewing* (SIHRB). Hops samples belonged to 12 hops varieties—each variety represented by an organic and a conventional sample. Among them, 11 organic–conventional hops samples were matched pairs, i.e., the same variety, country of origin and harvest year. The total number of hops samples was 28 (Supplementary material—Table S1).

In-house beer was produced from the samples of hops samples (*n* = 28). The amount of hop added was based on the predetermined content of α-acids –500 mg of α-acids in 1 L of wort. Beers were fermented using a bottom-fermented yeast strain SafLager W-34/70 (Fermentis, France) at 12 °C for five days to produce lager style beer. A blank matrix or “zero beer” was brewed according to the same procedure, only without the hops.

Commercial beer samples (*n* = 27, Supplementary material—Table S2) were purchased in Slovenia and Belgian stores. In this case, 15 different beers labelled as organic were matched with 12 conventional beers regarding style and alcohol content (±1%).

### 2.3. Sample Preparation

Hop pellets were ground in a mortar and sieved through a 0.5 µm sieve (No 35). A small quantity (0.1 g) of the sieved sample was then weighed in a falcon tube and extracted in 10 mL of methanol by sonication for15 min at room temperature. After centrifugation (5000 rpm for 5 min), the supernatants were filtered through a syringe filter and diluted in the injection solvent (0.1% FA in water/ACN, 70:30, *v/v*) in vials. The water content in the sieved samples was determined after heating 2 g of the sieved hops at 105 °C for 1 h.

### 2.4. Solid-Phase Extraction

Sample preparation began by extensive degassing the beer with consecutive hand mixing and sonication (for about an hour). The selected prenylflavonoids—XN, IXN and 8-PN, were extracted using solid-phase extraction (SPE) with Strata-X 33 µm polymeric reversed phase cartridge. The method is shown schematically in the Supplementary material—Figure S1. The cartridges were conditioned with 1 mL of 2% formic acid in water (Milli-Q) and equilibrated with 2 mL of water. Next, 950 mL of beer samples and 50 mL of methanol was loaded for the samples, while 950 mL of zero beer and 50 mL of spiked methanol was loaded for calibration and QCs. The washing step consisted of 2 mL of 40% methanol in water. After cartridges drying under vacuum (> 10” Hg, approx. 30 min), the analytes were eluted with 1 mL of 2% formic acid in methanol. The eluted samples were dried under nitrogen gas at 40 °C and reconstituted in the injection solvent, i.e., 1 mL of 0.1% formic acid in a water-acetonitrile mixture (30:70 *v/v*).

### 2.5. LC-MS/MS Method Development and Validation

The samples were analysed using liquid chromatography coupled to a tandem mass spectrometer system (LC-MS/MS) consisting of a Shimadzu Nexera X2 UHPLC (Kyoto, Japan) and QTRAP^®^ 4500 (Sciex, Framingham, MA, USA). Separation was achieved at 30 °C using an Ascentis^®^ Express C-18 column (50 × 2.1 mm, 1.7 μm) (Supelco, Bellefonte, PA, USA). The injection volume was 5 μL. The mobile phases consisted of 0.1% FA (A) and ACN (B). The gradient started with 30% B and increased to 70% in 1.5 min, followed by isocratic flow for 2.5 min, then fast return to initial conditions (30% B) in 0.1 min, and a final equilibration phase of 0.9 min. The total run time was 5 min, and the flow rate was 0.3 mL/min. The first 1.4 min of eluting solvent was sent to waste via the divert valve. The MS analyser was operated under electrospray ionisation (ESI) in negative mode with the following settings: curtain gas: 35 psi; source temperature: 200 °C; ion spray voltage: −4500 V; ion source gas one: 20 psi; and ion source gas two: 20 psi. Analyte quantification was performed in multiple reaction monitoring (MRM) mode using Analyst v1.6.3 software. All samples were injected in duplicate.

The matrix effect (ME) and extraction efficiency (EE) were calculated by applying the following equations:

(1) $$\mathrm{ME}\;\left(\%\right)=\frac{{\mathrm{Slope}}_{\;\mathrm{spiked}\;\mathrm{matrix}\;\mathrm{extract}}}{{\mathrm{Slope}}_{\mathrm{solvent}}}\times 100$$

(2) $$\mathrm{Extraction}\;\mathrm{efficiency}\;\left(\%\right)=\frac{{\mathrm{Slope}}_{\mathrm{pre}-\mathrm{extraction}\;\mathrm{spiked}\;\mathrm{matrix}}}{{\mathrm{Slope}}_{\mathrm{spiked}\;\mathrm{matrix}\;\mathrm{extract}}}\times 100$$

Calibration standards were prepared in duplicate by spiking matrices with a mixture of analytes at eight concentration levels: 0.5, 1, 5, 10, 50, 100, 500 and 1000 ng/mL. The validation matrix for hops was a hops pool, while the validation matrix for beer was zero beer. The method’s accuracy was determined by back-calculation using the regression equation (Eq. 4) and expressed as accuracy bias. The acceptable bias for the quality control (QC) of low, middle and higher concentration levels obtained for three replicates for each QC level was <15%. The low QC was considered the limit of quantification (LOQ).

(3) $$\mathrm{Bias}\;\left(\%\right)=\left(1-\frac{\mathrm{Calculated}\;\mathrm{concentration}}{\mathrm{Theoretical}\;\mathrm{concentration}}\right)\times 100$$

Precision was evaluated by performing a repeat analysis (*n* = 6) as intraday precision and over three days to estimate inter-day precision at the middle concentration level. Relative standard deviations were below 15%.

### 2.6. Isotope Ratio Mass Spectrometry (IRMS)

In bulk hops and beer samples (freeze-dried), the *δ*^13^C and *δ*^15^N values were determined using an IsoPrime100 isotope ratio mass spectrometer (IsoPrime, Cheadle, Hulme, UK) connected to a Vario PYRO Cube OH/CNS Pyrolyser/Elemental Analyzer (IsoPrime, Cheadle, Hulme, UK). Briefly, 2.5 mg of hop sample or 5 mg of beer samples were weighed into a tin capsule, closed with tweezers and put in the automatic sampler of the elemental analyser.

Stable isotope data are reported as deviations from an international standard and are given in the *δ*-notation (‰) using the general formula:

(4) δX*=[(X*X)sample(X*X)standard−1]×100

where δ^*^X refers to *δ*^13^C, *δ*^15^N, while ^*^X/X are the ratios ^13^C/^12^C, ^15^N/^14^N of the sample, and an international reference standard. Analyses were calibrated against the following international standards: USGS64 (glycine, *δ*^13^C = −40.81 ± 0.04‰) and USGS62 (caffeine, *δ*^13^C = −14.79 ± 0.04‰) and USGS62 (caffeine, *δ*^15^N = 20.17 ± 0.06‰) and IAEA-600 (caffeine, *δ*^15^N values of 1.02 ± 0.05‰). Other control reference materials were IAEA-600 caffeine with *δ*^13^C values of −27.73 ± 0.04‰ and CRP-IAEA casein with *δ*^13^C = −20.3 ± 0.09‰ for carbon and USGS64 (glycine) with *δ*^15^N values of 1.76 ± 0.06‰ and CRP-IAEA casein with *δ*^15^N values of 5.62 ± 0.19‰ for nitrogen. The *δ*^13^C values are reported relative to the V-PDB (Vienna-Pee Dee Belemnite) standard, while *δ*^15^N values are given relative to the V-CDT (Vienna Cañon Diablo Troilite) and AIR. Each sample was analysed in triplicate, and the means were calculated. The reproducibility for *δ*^13^C was ±0.2‰ and ±0.3‰ for *δ*^15^N.

### 2.7. Statistical Evaluation of the Data

Simple statistical analyses were carried out to check significant differences in measured parameters between conventionally and organically produced hops and beer using a Student t-test. Moreover, orthogonal projections to latent structures discriminant analysis (OPLS-DA) were performed on all the data to identify the most relevant parameters for discriminating hops and beer samples according to the production regime (organic/conventional).

## 3. Results and Discussion

### 3.1. Method Development and Validation

Sample preparation and analysis were based on literature methods [25,26] and optimised for our application. Supplementary material—Table S3 gives retention times and MRM parameters. Although several published methods [25,27,28] suggest filtering the samples using nylon filters, we found that these filters adsorbed the compounds of interest and that RC membrane filters were a better option.

Since there is no guideline for specifically analysing natural products in plants or foodstuffs,, our validation approach was based on AOAC [29], Eurachem guidelines [30], and other guidelines for analysing foodstuffs [31,32]. Different calibration approaches were tested, including solvent calibration (with and without an internal standard), matrix-matched calibration and standard addition calibration. Since stable isotope-labelled internal standards are not commercially available, structural analogues (surrogate standards), which are not present in the analysed samples (beer and hops), were tested as internal standards during the development stage. Accordingly, the flavanone eriodyctiol its chalcone analogue 3,4,2′,4′,6′-pentahydroxychalcone were tested as potential internal standards. Since there was no benefit in accuracy and precision from using these compounds as internal standards, the idea was discarded, while matrix-matched calibration was ruled out for hops because they contained the selected compounds. Therefore, standard addition calibration was used for the quantitative analysis of hops. Matrix-matched calibration, whereby the blank matrix was spiked before the extraction (also called modified matrix-matched calibration), was selected for beer, whereby zero beer was used as a blank matrix. The matrix was spiked before the extraction to compensate for the low and variable extraction efficiencies in both cases.

The validation results are presented in the Supplementary material—Table S4 and Table S5, respectively.

### 3.2. Hops and Beer Samples

The content of the selected analytes in hops samples (µg per gram of dry hops) are presented in Table 1. Since the samples contained a significant amount of water, concentrations were expressed on a dry weight basis. The water content in the sieved samples was determined after heating 2 g of the sieved hops at 105 °C for 1 h and is presented in Table 1.

**Figure 2:**
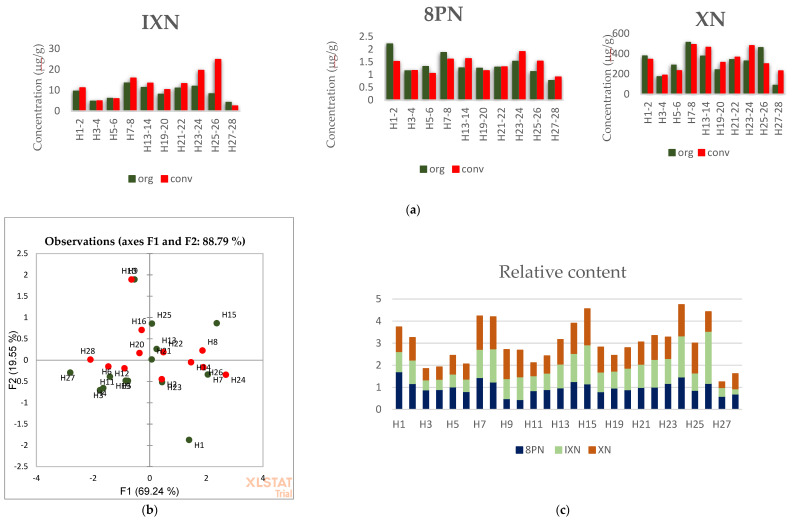
Histograms showing a comparison of the analyte content in the matched pairs of hops (**a**); PCA graph of hops samples with analyte content as variables (**b**); Histogram of total contents of the analytes in hops samples expressed as the sum of relative contents to the average level of a single analyte in the whole group (**c**).

The student t-test revealed no statistical difference in the content of the selected prenylflavonoids between the two groups: *p*-values were 0.3, 1 and 0.9 for IXN, 8PN and XN, respectively. A paired sample sign test comparing the differences in matched pairs also revealed no statistical difference, with *p*-values of 0.11, 0.75 and 0.75 for IXN, 8PN and XN, respectively (Figure 2a). Further, from the principal component analysis (PCA), it was impossible to discriminate between conventional and organic hop based on the prenylflavonoid content (Figure 2b). Figure 2c plots the relative contents of IXN, 8PN and XN. The relative contents were calculated as the amount of analyte in the sample/average amount of analyte in the sample group and were carried out to gain insight into the synthesis of the examined compounds in a sample, i.e., their relative ratios. Relative contents were chosen over their absolute contents in order to present the data on the same graph. The result revealed no strict proportionality in the compounds, i.e., transformations and metabolism, depend on many factors. The average concentrations were 340 ± 116 µg/g, 10.5 ± 5.0 µg/g and 1.3 ± 0.4 µg/g for XN, IXN and 8PN (*n* = 28), respectively. These results for the whole group of hop samples agrees with the published data. For example, Stevens et al. [25] obtained 478 µg/g, 8 µg/g and 2.1 µg/g for XN, IXN and 8PN (*n* = 1), respectively, while Magalhães et al. [28] reported 620 µg/g of XN, and IXN levels below the LOD (12 µg/g) in hop pellets samples (*n* = 1). De Keukeleire et al. [9] reported a XN content of 450 ± 215 µg/g.

**Figure 3:**
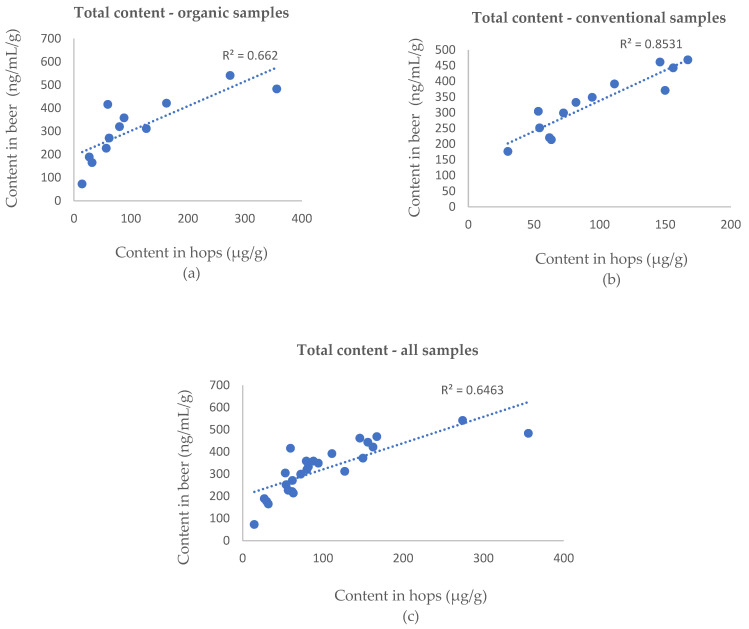
The total content of the selected prenylflavonoids in hops plotted against the total content of selected prenylflavonoids in beer in the (**a**), organic samples (**a**), conventional samples (**b**) and whole sample group (**c**).

Table 1 gives the concentrations of the prenylflavonoids in the in-house beer samples. The quantity of hop used for beer production was chosen according to its α-acids content (Supplementary material—Table S1). The total amount of the selected compounds in beer was expressed as a sum of the single analyte concentrations divided by the quantity of hop used in the brewing process (per gram of hops). The total content of IXN, 8PN and XN is expressed as the summed concentrations of the selected compounds in the whole hop samples, i.e., sample weight that includes water. The coefficients of determination (R^2^) in the total contents between hops and beer were 0.643, 0.662 and 0.853 for the whole group, organic samples and conventional samples, respectively (Figure 3). These findings suggest a linear correlation exists between the total content of the selected prenylflavonoids. The weaker correlations are likely due to metabolites with similar structures that play a role in transforming prenylflavonoids during the brewing process, which were not included in the study since they have no physiological activity. The weak correlation observed in organic samples likely reflects the diversity of similarly structured metabolites formed under less controlled conditions.

The *δ*^15^N and *δ*^13^C values in the hop samples are presented in Table 1. The *δ*^15^N values ranged from 5.3 to 10.9‰ for organic hops and are statistically higher (*p* = 0.027) than conventional ones (from 4.8 to 9.5‰). It is difficult to explain the *δ*^15^N values in hops since the processes and the fractionation influencing the N cycle is not well understood and hop N requirements can be satisfied from several sources. In addition to commercial fertiliser, soil organic matter, manure, cover crops and returned hop vines can supply substantial N for hop production and influence the *δ*^15^N values. It is interesting to note that the highest *δ*^15^N values in EU hops originate from the Czech Republic, one of the largest hop producers in the world (26). No statistically significant difference was observed in the *δ*^13^C values (average −26.7 ± 0.8‰) between conventionally and organically produced hops. However, *δ*^15^N values in European samples were lower than the organically produced hops [24].

### 3.3. Commercial Beer Samples

**Figure 4:**
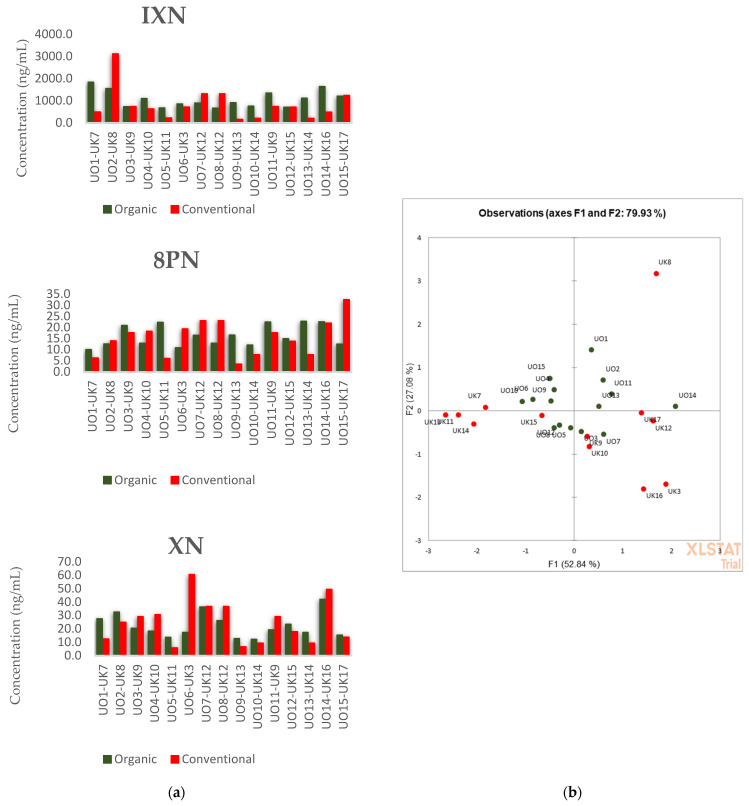
Comparison of the analyte content in the matched pairs of commercial beers (**a**); PCA graph of commercial beer samples with analytes contents as variables (**b**).

The levels of IXN, 8PN and XN in commercial beer samples are presented in Table 2. Student t-tests show no statistical difference in their content between the two groups, with *p*-values of 0.19 (IXN), 0.38 (8PN) and 0.33 (XN). The paired-sample sign test, comparing the differences in matched pairs, revealed no statistical difference with *p*-values (IXN = 0.6, 8PN = 0.1 and XN = 1, Figure 4a). Using the IXN, 8PN and XN content as a variable in the PCA did not discriminate between conventionally and organically grown hops (Figure 4b). Again, the levels of IXN, 8PN and XN in the whole group of beer samples (*n* = 27) agree with the published results with average concentrations of 0.023 ± 0.013 µg/L, 0.963 ± 0.593 µg/L and 0.016 ± 0.006 µg/L. Stevens et al. [25], investigating different beer styles and countries of production, obtained 0.023 ± 0.019 µg/L, 1.02 ± 0.98 µg/L and 0.049 ± 0.071 µg/L for XN, IXN and 8PN, respectively (*n* = 11).

The *δ*^15^N and *δ*^13^C values for commercial beer samples are presented in Table 2. The *δ*^15^N values for beers declared organic range from 3.4 to 7.4‰ and are statistically higher (*p* = 0.007) than non-organic beers (2.9 to 4.9‰). However, *δ*^15^N values are lower than those observed in organic hop samples. This value was expected since the hop represents only 1% of the beer components. Further, although the *δ*^15^N values were significantly higher in organically produced beer, it would be difficult to confirm the production regime based only on *δ*^15^N values since the conventional and organic beer data overlap. The *δ*^13^C values show no statistically significant difference between organic and non-organic beer, but the average *δ*^13^C value of −27.5 ± 0.6‰ indicate only C3 carbon. This finding is consistent with the previous studies performed on European beer. For example, Brooks et al. [17] found that European beer had an average *δ*^13^C value of −25.6 ± 1.5‰, indicating that only malt, a C3-source, was used during brewing as opposed to the presence of a C4-source in their composition, such as maise, which is typical for South and North American and Asian beers [18].

**Figure 5:**
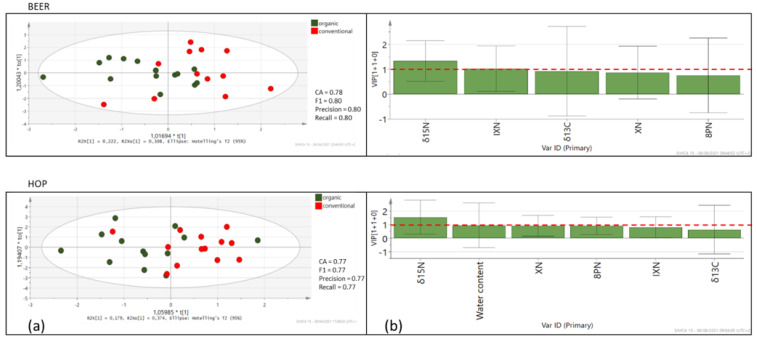
OPLS-DA score plots (**a**) with VIP values (**b**) in the pairwise comparison between different production practices for beer and hops. The red-dotted line at VIP = 1.0 indicates criteria for the identification of the most important model variable.

All data from the hop and beer were further statistically evaluated using OPLS-DA. The results are presented in Figure 5. In the OPLS-DA models, the leave-one-out was automatically used as cross-validation to obtain the misclassification result. The accuracy (CA), precision, recall and F1-score were calculated and presented for each model. The classification rate of these OPLS-DA models was between 77% and 80% for hops and beer, respectively, indicating a good separation between organic and conventional hops and beer despite the low number of samples. The variable importance in the projection (VIP) values of OPLS-DA models are also presented (Figure 5). A VIP value of more than 1.0 revealed that the corresponding variable was important in discriminating the production practice. In both cases, the *δ*^15^N value is the main discriminating parameter, while for beer, also IXN seems to be important. The evaluation of results needs to be confirmed by including a higher number of samples.

## 4. Conclusions

In conclusion, an LC-MS/MS method for analysing XN, IXN and 8PNwas developed and validated. Even though higher amounts of secondary metabolites in organically produced crops are reported in the literature, it was not the case in this study regarding hops. In addition, PCA based on the determined prenylflavonoid levels could not distinguish between organically and conventionally produced hops and beer. Instead, more likely, variety plays a more significant role in the synthesis of specific secondary metabolites than the agricultural management practice. In addition, the transformation of XN to IXN seems to be more complicated than schematically presented in the literature. Finally, measuring the level of XN, IXN and 8PN did not prove sufficient to detect mislabelling and other frauds. However, the *δ*^15^N values confirm previous findings that show statistically higher values in organic hops and beer. However, there is a potential overlap between the *δ*^15^N signatures, and it would not be easy to check the production type based solely on *δ*^15^N values. Despite this, separation could be further improved using OPLS-DA to give an overall correct classification rate of 77%. δ^15^N values and IXN were defined as the most promising parameters for differentiation between organically and non-organically produced beer. Even though obtained results are promising, further improvement of the methodology is needed, including analysing more samples from different years of production.

## Figures and Tables

**Table 1:** Stable isotope data and results of xanthohumol (XN), isoxanthohumol (IXN) and 8-prenylnaringenin (8PN) analysis of hop samples and in-house beer (*n* = 2).

Label	Type	δ^15^N (‰)	δ^13^C (‰)	Water Content (%)	Hops			Beer			
					IXN (μg/g)	8PN (μg/g)	XN (μg/g)	Weighed hops (g)	IXN (ng/mL)	8PN (ng/mL)	XN (μg/g)
H1	organic	7.9	−27.6	8.1	2.2	9.6	378	16.5	1030	19.8	263
H2	conventional	6.0	−27.1	7.0	1.5	11.2	345	24	1410	23.0	538
H3	organic	7.7	−27.1	8.0	1.1	4.7	173	27	553	17.2	296
H4	conventional	9.5	−27.1	9.0	1.2	4.9	188	35	671	18.4	363
H5	organic	6.6	−27.2	8.1	1.3	6.1	287	15	718	14.9	198
H6	conventional	7.5	−27.2	8.0	1.0	5.9	232	19	811	19.5	344
H7	organic	6.6	−27.7	8.4	1.9	13.5	511	5.8	1850	25.8	188
H8	conventional	6.2	−27.5	8.0	1.6	15.9	491	8.5	1120	19.5	283
H9	organic	5.3	−26.6	6.5	0.6	10.9	407	5.6	517	3.8	102
H10	conventional	8.1	−26.1	7.5	0.6	9.6	445	4.3	621	6.6	71.2
H11	organic	10.3	−26.9	8.7	1.1	7.1	199	27	644	8.2	79.2
H12	conventional	6.3	−26.6	8.1	1.2	7.9	264	21	1040	13.2	81.2
H13	organic	7.2	−25.5	8.0	1.3	11.4	376	18	1470	21.5	97.4
H14	conventional	4.8	−27.1	7.5	1.6	13.4	463	7.8	1130	16.9	66.9
H15	organic	7.5	−26.2	5.9	1.5	18.6	555	4.9	1290	14.2	38.6
H16	conventional	5.9	−26.8	5.9	1.0	9.4	384	8.5	1170	16.5	84.8
H19	organic	8.6	−26.2	9.9	1.3	8.1	242	11	570	11.5	44.4
H20	conventional	5.5	−26.4	8.0	1.2	10.3	314	9.6	617	13.4	65.6
H21	organic	10.9	−28.1	9.5	1.3	11.0	341	12	858	13.7	91.1
H22	conventional	5.9	−27.3	8.5	1.3	13.2	366	12	987	16.0	129
H23	organic	7.9	−26.9	8.5	1.5	11.9	327.3	7.7	896	17.5	67.1
H24	conventional	6.4	−27.0	8.0	1.9	19.6	480	8.2	1114.1	19.2	64.4
H25	organic	6.5	−25.8	11.4	1.1	8.3	460	11	595.6	6.5	54.5
H26	conventional	5.7	−25.6	7.0	1.5	24.8	301	5.2	196.1	5.0	76.0
H27	organic	5.4	−25.6	21.6	0.8	4.2	87.4	71	984.5	16.7	32.5
H28	conventional	5.4	−25.1	8.5	0.9	2.5	230	16	964.9	16.3	29.8

**Table 2:** Stable isotope data and results of the xanthohumol (XN), isoxanthohumol (IXN) and 8-prenylnaringenin (8PN) analysis in commercial beer samples (*n* = 2).

Label	Type	δ^15^N (‰)	δ^13^C (‰)	IXN (μg/L)	8PN (μg/L)	XN (μg/L)
UO1	organic	3.4	−27.2	1.834	0.010	0.027
UO2	organic	3.6	−27.0	1.549	0.012	0.032
UO3	organic	4.0	−27.4	0.727	0.020	0.020
UO4	organic	5.1	−28.3	1.096	0.012	0.018
UO5	organic	5.2	−25.9	0.676	0.022	0.014
UO6	organic	4.9	−28.4	0.856	0.011	0.017
UO7	organic	4.2	−27.4	0.894	0.016	0.036
UO8	organic	5.1	−27.2	0.662	0.013	0.026
UO9	organic	4.0	−27.9	0.907	0.016	0.013
UO10	organic	4.7	−27.9	0.754	0.012	0.012
UO11	organic	7.4	−26.7	1.35	0.022	0.019
UO12	organic	3.3	−27.9	0.705	0.015	0.023
UO13	organic	4.9	−28.4	1.11	0.023	0.017
UO14	organic	4.3	−27.9	1.64	0.022	0.042
UO15	organic	3.9	−27.9	1.20	0.012	0.015
UK3	conventional	4.1	−27.2	0.716	0.019	0.060
UK7	conventional	2.9	−28.3	0.492	0.006	0.012
UK8	conventional	4.6	−28.4	3.11	0.014	0.025
UK9	conventional	3.6	−27.4	0.740	0.017	0.029
UK10	conventional	3.3	−27.0	0.632	0.018	0.031
UK11	conventional	2.4	−27.4	0.221	0.006	0.006
UK12	conventional	3.3	−26.9	1.31	0.023	0.037
UK13	conventional	3.4	−27.3	0.155	0.003	0.007
UK14	conventional	3.2	−27.2	0.205	0.007	0.009
UK15	conventional	4.4	−27.2	0.713	0.013	0.018
UK16	conventional	4.9	−22.6	0.486	0.022	0.050
UK17	conventional	4.3	−27.5	1.24	0.032	0.014

## Supplementary Materials

The following are available online at https://www.mdpi.com/article/10.3390/foods10081839/s1, Figure S1: SPE protocol for sample preparation of beer samples, Table S1: Hops samples, Table S2: Beer samples, Table S3: Parameters of the LC-MS/MS method, Table S4: Validation data for hops, Table S5: Validation data for beer.
