# Supplementary material for: Differences in the Levels of the Selected Phytoestrogens and Stable Isotopes in Organic vs. Conventional Hops and Beer
Source: Foods. 2021 Aug 9;10(8):1839. doi: 10.3390/foods10081839 (PMC8394639; doi:10.3390/foods10081839)
Supplement: Supplementary file 1 [file foods-10-01839-s001.zip › foods-1278439-supplementary.pdf]

Table S1. Hops samples

Label	Variety	Harvest year	Country of origin	α-acids (%)	Seller
H1	BIO Saphir	2017	GER	3.03	Hopfen und mehr
H2	Saphir	2017	GER	2.1	Hopfen und mehr
H3	BIO Strisselspalter	2016	FRA	1.86	Hopfen und mehr
H4	Strisselspalter	2016	FRA	1.42	Hopfen und mehr
H5	BIO Tettnanger	2017	GER	3.36	Hopfen und mehr
H6	Tettnanger	2017	GER	2.64	Hopfen und mehr
H7	BIO Perle	2017	GER	8.66	Hopfen und mehr
H8	Perle	2017	GER	5.86	Hopfen und mehr
H9	BIO Chinook	2017	USA	8.95	Hopfen und mehr
H10	Chinook	2015	USA	11.56	Hopfen der Welt
H11	BIO HallertauerMittelfrüh	2016	GER	1.84	Hopfen der Welt
H12	HallertauerMittelfrüh	2017	GER	2.34	Hopfen der Welt
H13	BIO Perle	2016	GER	2.77	Hopfen der Welt
H14	Perle	2016	GER	6.4	Hopfen der Welt
H15	BIO Mandarina Bavaria	2016	GER	10.3	Hopfen der Welt
H16	Mandarina Bavaria	2017	GER	5.88	Hopfen der Welt
H19	BIO Hallertau Tradition	2017	GER	4.41	Hopsteiner
H20	Hallertau Tradition	2017	GER	5.2	Hopsteiner
H21	BIO Hallertau Select	2017	GER	4.24	Hopsteiner
H22	Hallertau Select	2017	GER	4.25	Hopsteiner
H23	BIO HallertauPerle	2017	GER	6.49	Hopsteiner
H24	HallertauPerle	2017	GER	6.08	Hopsteiner
H25	BIO Aurora	2017	SLO	4.65	SIHRB*
H26	Aurora	2017	SLO	9.62	SIHRB*
H27	BIO Celia	2017	SLO	0.7	SIHRB*
H28	Celia	2017	SLO	3.12	SIHRB*

**Slovenian Institute of Hop Research and Brewing*

Table S2. Beer samples

Organic beer samples					Conventional beer samples				
Label	Name	Style	Country	Alcohol (%)	Label	Name	Style	Country	Alcohol (%)
UO1	De Proefbrouwerij Nog eentje BIO	brown ale	BEL	6.4	UK3	Maister Brewery Gallus	pils	BEL	4.5
UO2	De Proefbrouwerij Nog eentje BIO	pale ale	BEL	6.4	UK7	St. Bernardus Pater 6	brown ale	BEL	6.7
UO3	Jessenhofke TRPL BIO	pale ale	BEL	8	UK8	De Ranke XX Bitter	pale ale	BEL	6
UO4	Belgoo BIO	pale ale	BEL	7.9	UK9	Waterloo Triple Blond	pale ale	BEL	8
UO5	Brasserie Lion BIO	pale ale	BEL	8	UK10	La Chouffe	pale ale	BEL	8
UO6	Brouwerij Strubbe Camille BIO	pils	BEL	5.2	UK11	Kasteel Blond	pale ale	BEL	7
UO7	Brouwerij StrubbeLeireken BIO	brown ale	BEL	6	UK12	Westmalle dubbel	brown ale	BEL	7
UO8	Jessenhofke BRWN BIO	brown ale	BEL	7	UK13	St. Bernardus Wit	pale ale	BEL	5.5
UO9	Dupont Blanche BIO	pale ale	BEL	5.5	UK14	Chimay blonde	pale ale	BEL	4.8
UO10	St-Feuillien Grisette BIO	pale ale	BEL	5.5	UK15	Andechser Spezial hell	pils	GER	5.9
UO11	Slaapmutske tripel BIO	pale ale	BEL	8	UK16	Waterloo Strong Dark	brown ale	BEL	8
UO12	Pinkus Special	pils	GER	5.1	UK17	Duvel	pale ale	BEL	8.5
UO13	Puur La Trappe BIO	pale ale	NED	4.7					
UO14	Brouwerij Strubbe Alfoncine BIO	pale ale	BEL	8.5					
UO15	Gageleer BIO	pale ale	BEL	7.5					

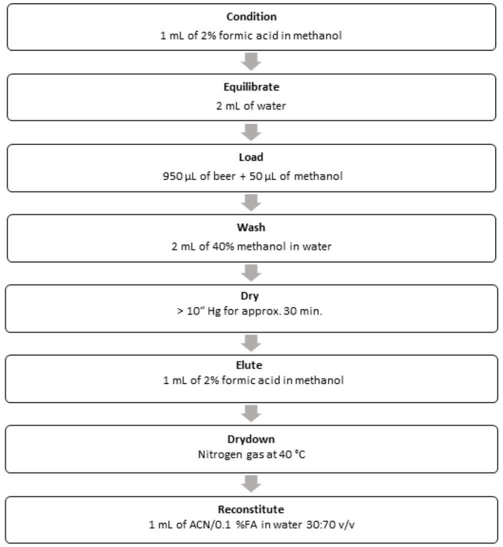

Figure S1. Figure 2: SPE extraction procedure

Table S3. Parameters of the LC-MS/MS method

**Analyte**	**Retention time (min)**	**Quantifier transition**	**Qualifier transition**	**CE (eV)**	**CXP (V)**
**IXN**	1.90	353 → 233	353 →119	-26	13
**8PN**	2.50	339 → 219	339 →119	-30	7
**XN**	3.25	353 → 233	353 →119	-26	13

Table S4. Validation data for hops

Analyte	Regression	R^2	Range (ng/ml)	Precision intra-day (RSD)			Precision inter-day (RSD			Accuracy (Bias)			Matrix effect	Extraction recovery
				QC1	QC2	QC3	QC1	QC2	QC3	QC1	QC2	QC3		
**IXN**	y=6982.8x+9585	0.9998	10 - 100	5%	2%	5%	6%	2%	5%	+1%	+6%	**+8%**	77%	95%
**8PN**	y= 33113x-8750.1	0.9999	0.5 - 1000	6%	2%	4%	5%	4%	4%	-13%	0%	+4%	99%	97%
**XN**	y=5476.2x+40866	0.9998	10 - 1000	13%	8%	12%	10%	11%	1%	-3%	+2%	-1%	98%	70%

Table S5. Validation data for beer

Analyte	Regression	R^2	Range (ng/ml)	Precision intra-day (RSD)			Precision inter-day (RSD			Accuracy (Bias)			Matrix effect	Extraction recovery
				QC1	QC2	QC3	QC1	QC2	QC3	QC1	QC2	QC3		
**IXN**	y=12882x+68928	0.9995	2.5 - 100	4%	2%	1%	4%	2%	2%	-11%	+5%	-1%	102%	74%
**8PN**	y= 57461x-51633	0.9999	1 - 100	3%	2%	2%	5%	2%	1%	+12%	-4%	0%	105%	62%
**XN**	y=20745x+27148	0.9998	5 - 500	4%	1%	1%	3%	1%	1%	+9%	+5%	0%	93%	78%
